# Supplementary material for: Seminoma and Embryonal Carcinoma Footprints Identified by Analysis of Integrated Genome-Wide Epigenetic and Expression Profiles of Germ Cell Cancer Cell Lines
Source: PLoS One. 2014 Jun 2;9(6):e98330. doi: 10.1371/journal.pone.0098330 (PMC4041891; doi:10.1371/journal.pone.0098330)
Supplement: File S1 — ZIP file containing DMRforPairs output for significant regions. Please start from the html files. (ZIP) [file pone.0098330.s008.zip › figures/25843.pdf]

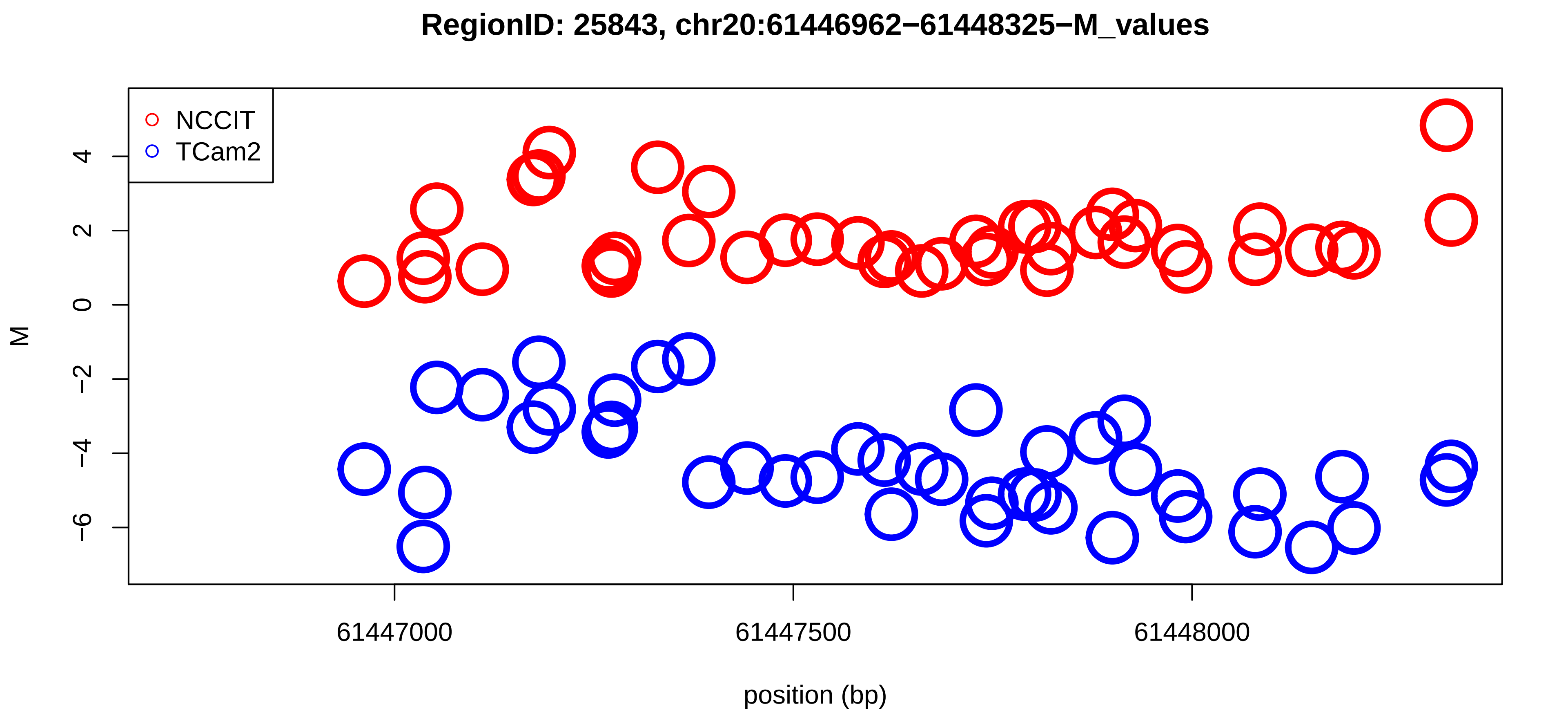

RegionID: 25843, chr20:61446962−61448325−M_values
NCCIT
TCam2
M
4
2
0
−2
−4
−6
61447000
61447500
61448000
position (bp)

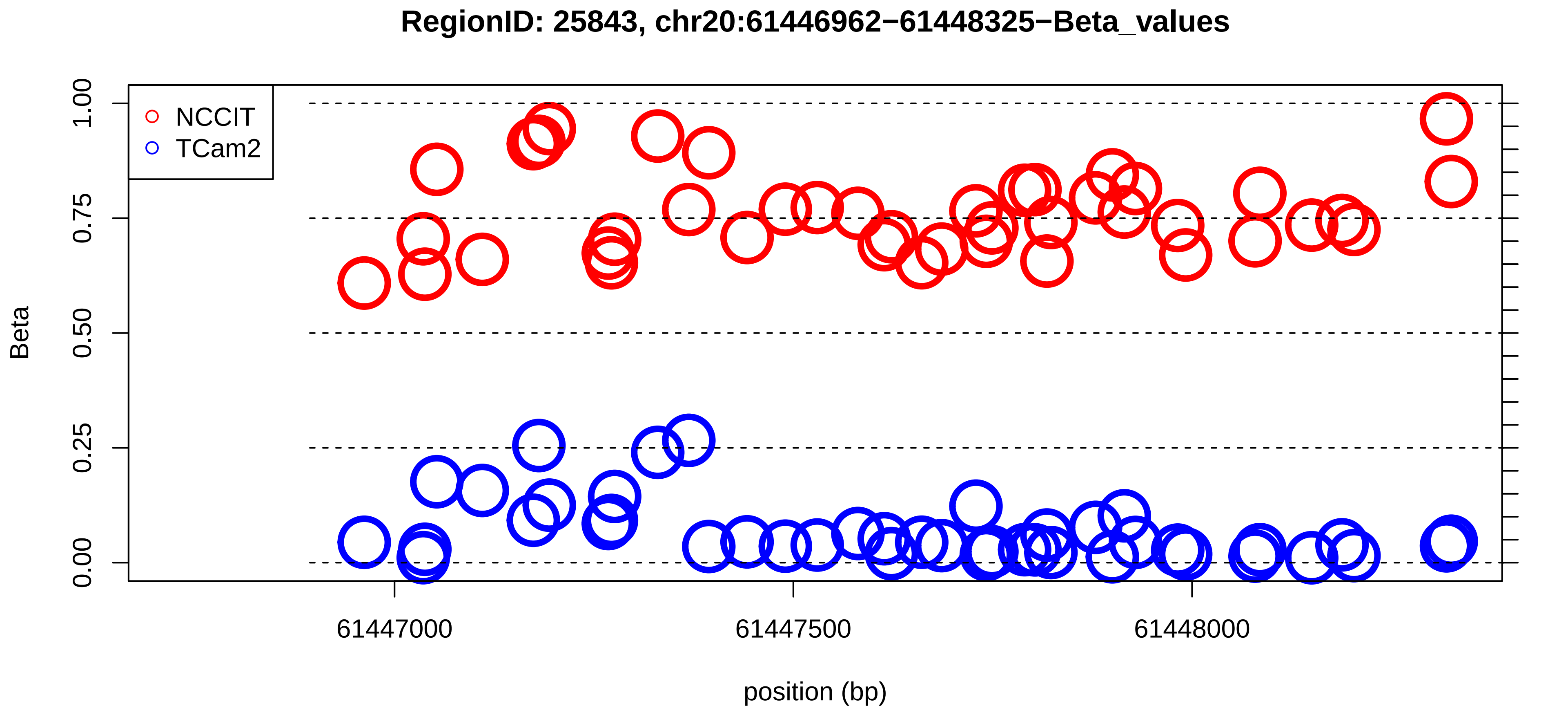

RegionID: 25843, chr20:61446962−61448325−Beta_values
NCCIT
TCam2
Beta
1.00
0.75
0.50
0.25
0.00
61447000
61447500
61448000
position (bp)

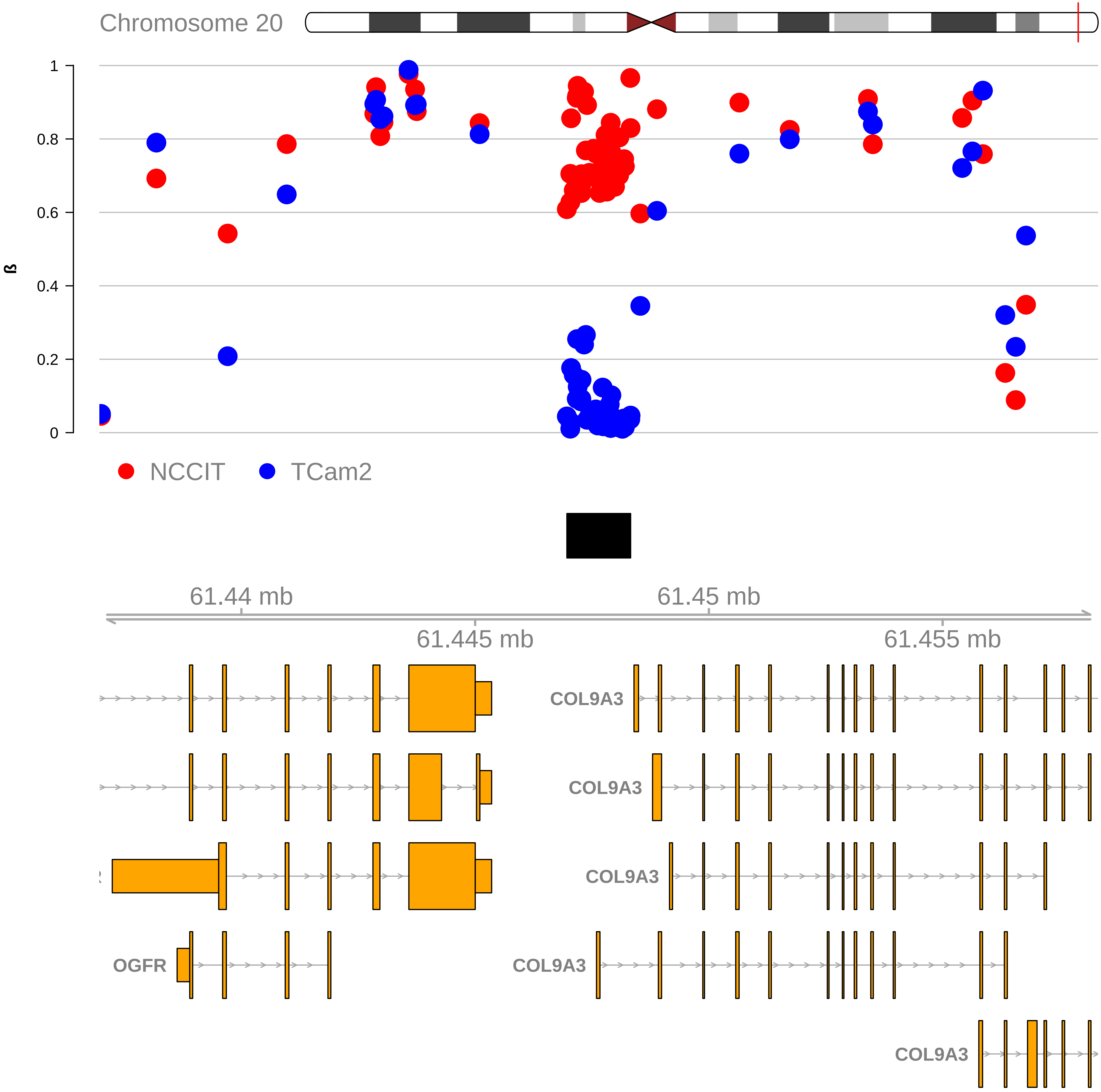

Chromosome 20
1
0.8
0.6
0.4
0.2
0
β
NCCIT
TCam2
61.44 mb
61.45 mb
61.445 mb
61.455 mb
COL9A3
COL9A3
COL9A3
OGFR
COL9A3
COL9A3
